# Small Bowel Obstruction Masquerading as Acute ST Elevation Myocardial Infarction

**DOI:** 10.1155/2015/685039

**Published:** 2015-10-26

**Authors:** Manan Parikh, Martin Miguel Amor, Isha Verma, Jeffrey Osofsky, Madhu Paladugu

**Affiliations:** ^1^Department of Internal Medicine, Monmouth Medical Center, 300 2nd Avenue, Long Branch, NJ 07740, USA; ^2^Department of Cardiology, Monmouth Medical Center, 300 2nd Avenue, Long Branch, NJ 07740, USA

## Abstract

ST segment elevation on EKG remains among the most important presentations of acute myocardial infarction. Due to the urgency of intervention for this finding, other clinical scenarios causing ST elevations on EKG may sometimes go unaddressed and can lead to fatal complications. We present a case of an 86-year-old male presenting with small bowel obstruction leading to EKG findings of ST segment elevation in the absence of critical coronary obstruction. The EKG finding resolved after the improvement of small bowel obstruction reflecting the reversible cause of the changes.

## 1. Introduction

ST segment elevation on EKG remains among the most important presentations of acute myocardial infarction, requiring immediate diagnosis and management to prevent permanent damage to the myocardium and reduce mortality. Due to the urgency of intervention for this finding, other clinical scenarios causing ST elevations on EKG may sometimes go unaddressed and can lead to fatal complications. Herein, we present an unusual case of an 86-year-old male with ST segment elevations on EKG, which while initially thought to be cardiac were later found to be due to small bowel obstruction.

## 2. Case Report

**Figure 1:**
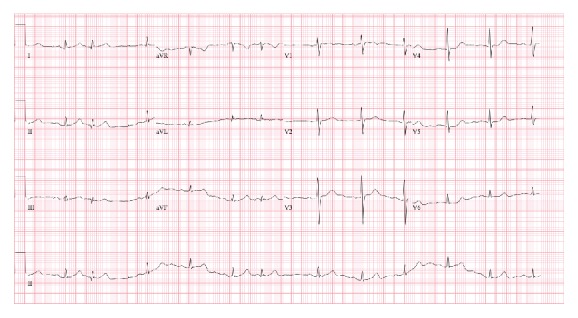
12-lead EKG on admission showing normal sinus rhythm.

**Figure 2:**
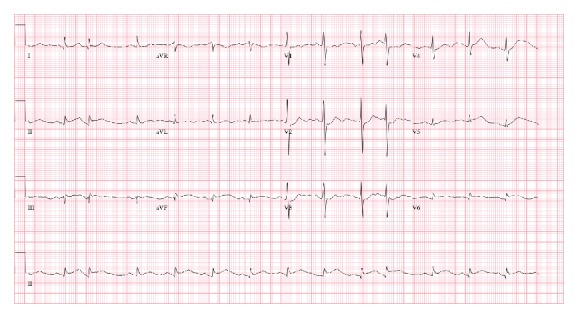
12-lead EKG showing ST segment elevations in leads II, III, and aVF with reciprocal changes in anterior precordial leads, consistent with acute inferior wall ST segment elevation myocardial infarction (STEMI).

**Figure 3:**
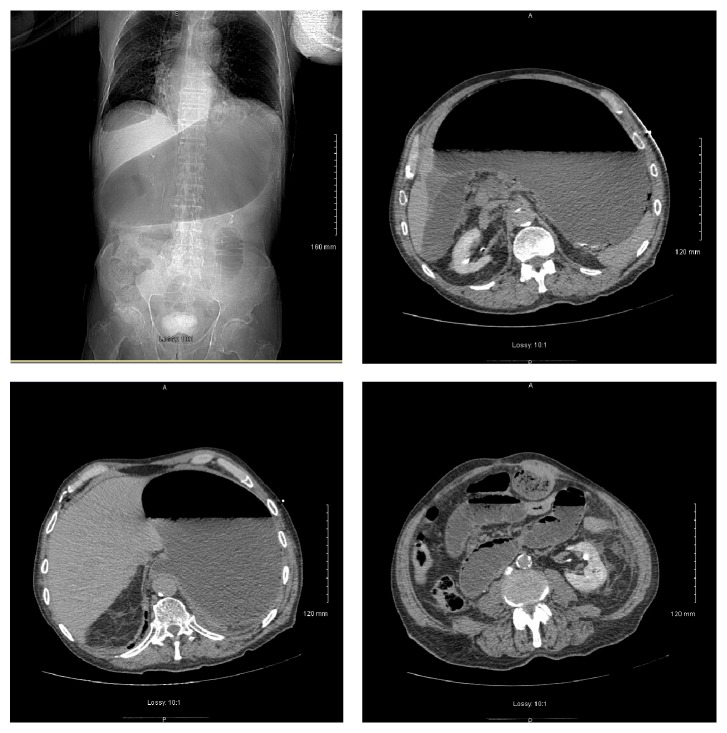
CT scan of the abdomen showing small bowel obstruction, with transition point in the jejunum with a markedly dilated stomach containing foci of air. Also a dilated gall bladder with gallstones and normal-appearing pancreas is noted.

**Figure 4:**
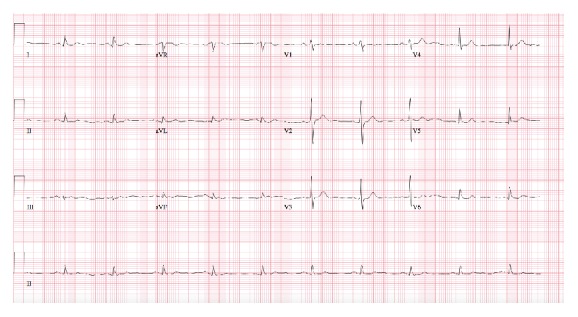
12-lead EKG showing normal sinus rhythm, with resolution of the previously seen ST segment elevations in the inferior wall leads (II, III, and aVF).

An 86-year-old male presented to the ED with a chief complaint of vomiting and epigastric pain of 24-hour duration. He had a past medical history significant for paroxysmal atrial fibrillation, hypertension, chronic kidney disease, anemia of chronic disease, Barrett's esophagus, esophageal rupture, and pneumomediastinum requiring surgical repair. Ten years prior to this ED visit, the patient had undergone an emergency esophageal rupture repair with prolonged postsurgical course requiring jejunostomy and tracheostomy but had subsequently recovered from it. He had a nuclear stress test 10 months prior to this visit with no signs of inducible ischemia. He started experiencing epigastric abdominal pain as well as nausea 24 hours prior to presentation. In the following hours, he had three episodes of vomiting and one bowel movement. There was no note of any hematemesis, hematochezia, or melena. Upon arrival at the ED, his vital signs were stable with blood pressure of 166/70 mmHg, pulse rate of 83, respiratory rate of 14, and temperature of 98.2 F. His initial hemoglobin was 11.0 mg/dL. He also had elevated amylase and lipase levels of 198 IU/L and 76 IU/L, respectively, which increased to 474 IU/L and 527 IU/L the next morning. Abdominal obstructive series revealed scant small bowel gas without clear evidence of obstruction. Ultrasound of the abdomen showed a large gallstone in common bile duct with no gall bladder wall thickening or pericholecystic fluid. An EKG obtained at this time showed sinus rhythm with no ST segment elevation (Figure 1). He was admitted as a case of acute pancreatitis. On the succeeding hospital day, he was noted to have severe epigastric pain and another EKG was obtained. The EKG (Figure 2) showed ST segment elevations in inferior leads with reciprocal changes in anterior precordial leads consistent with acute inferior wall ST segment elevation myocardial infarction (STEMI). Troponin was noted to be 0.04. Emergent cardiac catheterization revealed 70% stenosis of the right coronary artery (RCA), as well as diffuse calcific disease of posterolateral branches, with 40% left anterior descending artery stenosis. None of the vessels showed plaque rupture or acute thrombus. Serial monitoring of troponin levels yielded normal results. In light of the patient's ongoing gastrointestinal complaints, intervention for the noncritical RCA lesion was deferred. The patient subsequently underwent an abdominal CT scan and was found to have small bowel obstruction, with transition point in the jejunum with a markedly dilated stomach containing foci of air (Figure 3). He also had a dilated gall bladder with gallstones, but the pancreas was normal in appearance. Small bowel obstruction and gastric distention were conservatively treated with nasogastric tube placement over the next 2 days. As the patient's gastric distention improved, the inferior wall ST segment elevations in his EKG also resolved, as was noted on serial EKGs (Figure 4). The patient subsequently underwent laparoscopic cholecystectomy without complications. He was eventually discharged home with stable follow-up.

## 3. Discussion

Despite the variety of diagnostic tests available, the EKG remains the primary diagnostic tool to diagnose acute myocardial infarction. Hence, it is important to know about conditions which can present with ST elevations on EKG. Other than myocardial infarction, common cardiac conditions causing ST elevations on EKG include myocarditis [1, 2], early repolarization, ventricular hypertrophy, and aneurysms [3]. Other noncardiac conditions, which may present with ST elevations on EKG, include cholecystitis [4], esophageal perforation [5], pancreatitis [6], and stomach distention [7]. These are usually misdiagnosed as acute myocardial infarction resulting in emergent angiography and unwanted thrombolytic therapy for early reperfusion. The ST segment elevations seen on EKG in these noncardiac conditions may be explained by disease processes involving the intrathoracic cavity, producing a shift in the main QRS axis of the heart as a result of displacement of the intrathoracic contents. Specifically, these EKG changes occur secondary to displacement of the heart in the anterior-posterior plane, or due to the thickness of the contents between the chest wall and heart [8].

In our patient, distention of the stomach and esophagus secondary to small bowel obstruction possibly caused changes in the relative position of the heart to the other thoracic organs. This presented on EKG in the form of ST elevations in leads II, III, and aVF, which was mistaken for acute inferior wall myocardial infarction. Subsequently, the patient was taken for immediate angiography, but no critical lesion was found. Appropriate intervention to relieve small bowel obstruction resulted in improvement of the symptoms and resolution of the EKG changes.

In conclusion, while the EKG remains an indispensable tool to diagnose acute myocardial infarction, it is important to rule out other cardiac conditions presenting with ST elevations on EKG before pursuing aggressive interventions. It is equally important to be cognizant of noncardiac conditions presenting with ST segment elevations on EKG, to guide us in choosing the most appropriate intervention. With this case report, we intend to add small bowel obstruction to the growing list of noncardiac conditions presenting as ST elevations on EKG.
